# Whole Blood Transcriptomics and Urinary Metabolomics to Define Adaptive Biochemical Pathways of High-Intensity Exercise in 50-60 Year Old Masters Athletes

**DOI:** 10.1371/journal.pone.0092031

**Published:** 2014-03-18

**Authors:** Kamalika Mukherjee, Brittany A. Edgett, Harrison W. Burrows, Cecilia Castro, Julian L. Griffin, Adel Giaid Schwertani, Brendon J. Gurd, Colin D. Funk

**Affiliations:** 1 Department of Biomedical and Molecular Sciences, Queen's University, Kingston, Ontario, Canada; 2 School of Kinesiology and Health Studies, Queen's University, Kingston, Ontario, Canada; 3 Department of Biochemistry and the Cambridge Systems Biology Centre, University of Cambridge, Cambridge, United Kingdom; 4 Division of Cardiology, McGill University Health Centre, Montreal, Quebec, Canada; Instituto de Investigación Sanitaria INCLIVA, Spain

## Abstract

Exercise is beneficial for a variety of age-related disorders. However, the molecular mechanisms mediating the beneficial adaptations to exercise in older adults are not well understood. The aim of the current study was to utilize a dual approach to characterize the genetic and metabolic adaptive pathways altered by exercise in veteran athletes and age-matched untrained individuals. Two groups of 50–60 year old males: competitive cyclists (athletes, n = 9; VO_2peak_ 59.1±5.2 ml·kg^−1^·min^−1^; peak aerobic power 383±39 W) and untrained, minimally active individuals (controls, n = 8; VO_2peak_ 35.9±9.7 ml·kg^−1^·min^−1^; peak aerobic power 230±57 W) were examined. All participants completed an acute bout of submaximal endurance exercise, and blood and urine samples pre- and post-exercise were analyzed for gene expression and metabolic changes utilizing genome-wide DNA microarray analysis and NMR spectroscopy-based metabolomics, respectively. Our results indicate distinct differences in gene and metabolite expression involving energy metabolism, lipids, insulin signaling and cardiovascular function between the two groups. These findings may lead to new insights into beneficial signaling pathways of healthy aging and help identify surrogate markers for monitoring exercise and training load.

## Introduction

Regular exercise has well-known health benefits and mitigates many of the devastating consequences of age-related disorders including cardiovascular disease, diabetes, obesity, cancer, dementia, anxiety and depression [1]. While these beneficial effects are accomplished through exercise mediated adaptation at physiological, anatomical and biochemical levels, the molecular mechanisms mediating these adaptations are still not completely understood.

In particular, limited evidence exists regarding the systemic genetic and metabolic adaptations to prolonged vigorous-intensity exercise in older individuals. While the effect of acute exercise on gene expression has been studied in young inactive adult men [2]–[5], custom-made cDNA microarrays probing a small specific subset of stress- or immune-response genes have also been utilized to examine the effect of exercise in young athletes [6], [7]. However, an extensive systems biology approach towards understanding the genetic, as well as metabolic variations between well-trained veteran athletes and their age matched untrained counterparts is still lacking.

The majority of gene expression studies related to exercise in humans have used either skeletal muscle or peripheral blood mononuclear cells (PBMCs), which represent only a fraction of white blood cells (WBCs) present in whole blood [4], [8], [9]. WBCs are known to express numerous genes, whose expression levels are modified by genetic and environmental factors [3]. Moreover, ∼80% of the blood transcriptome has been reported to be shared with various tissues of the body including the heart, brain, kidney and lungs [10]. This indicates that subtle changes occurring in individual systems throughout the body can leave genetic “footprints” in blood, suggesting that peripheral blood has the potential to be used as an important diagnostic tool for a variety of pathophysiological conditions. In addition, various metabolomic studies have utilized human plasma to elucidate the effects of exercise on metabolite expression in untrained healthy men as well as professional athletes [11], [12]. Recent studies examining exercise-induced changes in the metabolic profile of urine suggest that the urine metabolome may also provide valuable insight into whole body physiological function [13], [14].

Altogether, this led us to hypothesize that well-trained veteran athletes may possess a unique genetic/metabolic signature in response to exercise when compared to untrained age-matched individuals. In the current study, we used a dual approach to identify these potential differences in genetic, biochemical and metabolic signaling pathways in whole blood and urine samples, utilizing genome-wide DNA microarray analysis and NMR spectroscopy-based metabolomic profiling, respectively. Thus, our overall aim was to identify the differences in gene and metabolite expression in trained versus untrained older adults in response to exercise. Our results provide new insights into signaling pathways that may contribute to the beneficial effects of exercise and provide a starting point as we continue to build towards a better understanding of healthy aging.

## Materials and Methods

### Ethics Statement

The Members of the Queen's University Health Sciences & Affiliated Teaching Hospitals Research Ethics Board have examined the research protocol, medical clearance form and the revised consent form for your project and consider it to be ethically acceptable.

### Subjects

Seventeen healthy men between the ages of 50 and 60 years were recruited for the study and assigned to one of two groups: athlete (trained) or control (untrained). The subjects in the trained group included 9 Masters level athletes involved regularly in competitive cycling (1 triathlete) competition (>5 hours per week of training including regular high-intensity intervals, range: 5-18 hours per week) with at least 8 years of competitive racing (road, mountain and cyclocross) with most considered lifelong athletes (range: 8–42 years) among which were national, provincial and regional age group champions or podium winners). The untrained group included 8 individuals who were minimally active (0.5–3 hours per week of low-intensity exercise) and were not involved in any training program. Males were selected for the study primarily due to recruitment issues involving scarcity of older female competitive cyclists in the Kingston area, as well as to exclude complications arising from gender biases and the effect of the menstrual cycles on metabolic and stress/inflammatory responses [15]. Other exclusion criteria included any history of cardiovascular or metabolic diseases, use of prescription or recreational drugs, and smokers. The experimental protocol and associated risks were explained to all the participants before written consent was obtained. The study protocol was approved by the Health Sciences Research Ethics Board at Queen's University.

### Study Design

Participants were required to visit the laboratory on three separate occasions. Twenty-four hours prior to each visit, participants were asked to refrain from any alcohol consumption or any form of exercise outside of the study intervention. During the first visit anthropometric measures (height, weight, BMI and waist circumference) were recorded, and the subjects were asked to complete a peak oxygen consumption (VO_2peak_) test (described below).

A minimum of 2 days, but less than 7 days following the first visit each participant was provided with a standardized dinner (Stouffer's Sauté Sensations; 520 kcal; 37 g carbohydrate (CHO), 5 g fat, 16 g protein), a fruit cup (Dole; 160 kcal; 30 g CHO, 3.5 g fat, 2 g protein), and 500 ml of 2% milk (260 kcal; 12 g CHO, 5 g fat, 9 g protein) to be ingested at about 19:00 h, but no later than 21:00 h the night before reporting to the lab for their second visit. Following a subsequent 12 h fast, participants reported to the lab at 09:00 h the following morning. Upon arrival, participants were provided with a small standardized breakfast consisting of a plain bagel (190 kcal; 36 g CHO, 1 g fat, 7 g protein), ∼15 g of cream cheese (45 kcal; 1 g CHO, 4 g fat, 1 g protein), and 200 ml of apple juice (90 kcal; 22 g CHO, 0 g fat, 0 g protein). Fifteen minutes following breakfast, a submaximal endurance test was performed (described below).

### VO_2peak_ Test

For all exercise protocols the trained group used their own race bicycles fitted to a RacerMate CompuTrainer (Seattle, WA), while the untrained group utilized a cycle ergometer (Monark, Ergomedic 874E, Varberg, Sweden). For the trained group, the CompuTrainer was calibrated before the VO_2peak_ test and submaximal endurance ride as per the manufacturer's instructions (RacerMate Computrainer, Seattle, WA). The calibration process also served as a warm-up for the trained group. For the VO_2peak_ test, gas exchange was measured using a metabolic cart (Moxus, AEI Technologies, Pittsburgh, PA). Trained participants performed the ramp test with an increase of 25 W/min until the participant reached volitional fatigue. Untrained participants initially cycled load-less (∼25 W) at a cadence of 80 rpm for 6 min, followed by a step increase to 80 W for 1 min. After the step increase to 80 W, work rate (WR) was similarly increased by 25 W/min until participants also reached volitional fatigue. Volitional fatigue was determined by the inability of the participant to maintain a minimum cadence of 60 rpm. VO_2peak_ was calculated using the highest 30 sec average VO_2_ during the final stage of the ramp protocol and WR_peak_ was calculated as the highest WR achieved during the test.

### Submaximal Endurance Ride

A submaximal endurance ride was performed on the second visit and involved participants' cycling on the same bicycle or cycle ergometer used for their respective VO_2peak_ test. Participants performed 6 min of cycling at ∼25 W and at a cadence of 80 rpm, followed by a 45 min ride at 60% of each individual's peak work rate (WR_peak_). The test was then ramped up to 90% of WR_peak_ and ended when participants reached volitional fatigue.

### Blood and Urine Collection

Blood was withdrawn from the antecubital vein of 9 athletes and 7 control subjects (blood could not be withdrawn from one of the control subjects) immediately prior to consuming breakfast (time-point T1; pre-exercise), immediately following the submaximal endurance ride (time-point T2; immediate post-exercise sample), and 24 h following the submaximal endurance ride (time-point T3; 24 h post-exercise). Blood from time-point T1 (pre-exercise) was stored in multiple tubes for RNA extraction, and for measurements of fasting blood glucose, insulin, lipid profile, hematocrit and complete blood counts. Blood from time-points T2 (immediately post exercise) and T3 (24 h post exercise) were stored for RNA extraction. Participants were also provided with urine collection jugs and instructed to collect urine for 24 hours prior to the submaximal endurance ride visit (pre-exercise 24 hour urine sample) and for 24 hours following the submaximal endurance ride (post-exercise 24 hour urine sample). The pre-exercise urine sample was collected by researchers at time-point T2 and the post-exercise sample at time-point T3. All urine samples were inverted 2–3 times, and 10 ml aliquots were placed into 15 ml tubes. Sodium azide was then added (0.01% wt/vol final) and samples were stored at −80°C until further analysis.

### Blood RNA Preparation

Whole blood samples for each participant were stored in PAXgene blood RNA tubes (Qiagen GmbH, Germany) at −80°C from all three time-points until further analysis. RNA was isolated from blood sample aliquots (2.5 ml) using a PAXgene blood RNA kit (Qiagen GmbH, Germany) according to the manufacturer's protocol. Residual DNA was removed with DNase I treatment, according to the manufacturer's instructions (Qiagen GmbH, Hombrechtikon, Germany). The quality of the isolated RNA was checked with Agilent RNA 6000 Nano Assay kit reagents using an Agilent 2100 Bioanalyzer (Agilent Technologies, Santa Clara, CA, USA). Samples with a RNA Integrity Number (RIN) of 7.0 or higher were used for further analysis. As a result of this, one athlete sample at time-point T2 was discarded. In addition, 4 samples at each of T1 and T3 time-points were lost inadvertently due to cracked tubes and a freezer incident. The RNA samples were quantified using a standard Nanodrop ND-1000 spectrophotometer (Nanodrop, Wilmington, DE, USA).

### RNA Labeling and Microarray Hybridization

Total RNA (200 ng) from each sample was spiked with One-Color RNA Spike-In Kit reagents (Agilent Technologies, Santa Clara, CA, USA) according to the manufacturer's instructions. The spike mix contains ten *in vitro* synthesized, polyadenylated transcripts in predetermined ratios, which hybridize with specific control probes on the Agilent microarray and provide readily accessible data for quality control of sample hybridization. Subsequent amplification and labeling of the samples to generate fluorescent cRNA (complimentary RNA) were carried out using the One-Color Quick Amp Labeling Kit (Agilent Technologies, Santa Clara, CA, USA) according to the manufacturer's instructions. The method uses T7 RNA polymerase, which simultaneously amplifies target material and incorporates cyanine (Cy) 3-labeled CTP. Labeled cRNA was purified using an RNeasy Mini kit (Qiagen). Only cRNA samples with a yield >1.65 μg, and a specific activity >9 pmol Cy3/μg cRNA were used for subsequent hybridization onto microarray slides.

cRNA samples were hybridized to Agilent Whole Human Genome (4 × 44 K) Microarrays (Agilent Technologies, Santa Clara, CA, USA) for 17 h at 65°C. These arrays contained multiple probe sets for each gene in order to enhance confidence of the hybridization data. Arrays were subsequently washed according to the manufacturer's instructions and scanned using an Agilent Microarray Scanner (Agilent Technologies, Santa Clara, CA, USA). The resultant data were feature-extracted using Feature Extraction Software 10.5.1.1 (Agilent Technologies, Santa Clara, CA, USA) and checked for quality control. The data discussed in this manuscript has been deposited in NCBI's Gene Expression Omnibus (GEO) and are accessible through GEO Series accession number GSE51216.

### Microarray Data Analysis

Raw data obtained from the microarrays were imported into Nexus Expression 3.0 (BioDiscovery Inc, Hawthorne, CA, USA) and processed according to the default settings of the software, which included a local background correction, removal of flagged spots and performing quantile normalization. The samples were thereafter assigned different factors based on the type (control/untrained or athlete/trained), or the time-points they belonged to (T1, T2 and T3). A filtering setting was set to remove probes with variance greater than 0.1 across the selected samples. Differentially regulated probes/genes were identified across the two groups at the three time-points with a minimum threshold of 1.5-fold change, statistical significance set at *p*-value<0.05, and a Benjamini-Hochberg multiple testing correction with false discovery rate (FDR) at less than 0.1. Using an empirical Bayesian approach, an intensity-based pooling that involved 200 probes was used to improve variance estimate and add significance to the data-set. Heatmaps representing the expression of probes across samples were generated and clustered based on the default parameters of the software. These included clustering of samples based on an average hierarchical algorithm and Euclidean metrics, while probes were clustered based on an average hierarchical algorithm and Pearson correlation metrics. A set of probes greater than 1000 in number was clustered by k-means algorithm (cluster count 5) and Pearson correlation metrics. In addition, GeneSpring GX 12.1 software (Agilent Technologies, Santa Clara, CA, USA) was also used with the default settings of the manufacturer to validate the results obtained from the Nexus Gene Expression 3.0 analysis.

### Real-Time Quantitative PCR

Total RNA (500 ng) was reverse-transcribed using an iScript cDNA synthesis kit (Bio-Rad, Hercules, CA, USA) according to the manufacturer's instructions. The resultant cDNA was subjected to quantitative real-time polymerase chain reaction (qRT-PCR) using Taqman gene expression assays (Applied Biosystems, Foster City, CA, USA). To account for variability in the starting material between samples, messenger RNA (mRNA) encoding the housekeeping gene hypoxanthine phosphoribosyl transferase (HPRT) was quantitated for an internal normalizing control. All primer/probe assays used in the current study were purchased from Applied Biosystems. Results were represented as fold change after calculations using the delta-delta Ct method. Statistical significance was determined using an unpaired t-test with GraphPad Prism software (La Jolla, CA, USA) and differences were considered statistically significant at *p*<0.05.

### ELISA

Urotensin concentrations in blood were measured using an Urotensin II ELISA (human) ELISA kit (Promokine, Germany) following the manufacturer's instructions. The plasma was diluted 1∶10 in the assay buffer provided by the kit, and absorbance was read at 450 nm against 620 nm as reference.

### NMR Spectroscopy Acquisition

Aliquots of 300 μl urine samples were made up to 500 μl with phosphate buffer (0.2 M, pH 7.4) and 100 μl of sodium 3-trimethylsilyl-(2,2,3,3-^2^H_4_)-1-propionate (TSP)/D_2_O/sodium azide solution (0.05% wt/vol TSP in D_2_O and 1% wt/vol sodium azide). Proton NMR data acquisition was performed on a Bruker 500 MHz instrument (Bruker, Karlsruhe, Germany) using a 5 mm broadband probe with the sample maintained at 27°C. One dimensional NOSEY presaturation spectra were collected for pre- and post-exercise samples into 32,000 data points with a spectral width of 8000 Hz over an acquisition time of 4.1 s and averaged over 128 scans. Spectra were Fourier transformed with a line broadening factor of 1 Hz and the chemical shift of the spectra referenced to TSP manually. All processing to this stage was performed using Topspin (version 2, Bruker, Karlsruhe, Germany).

### Spectral Reduction and Multivariate Statistical Analysis

NMR spectra were phased and auto-baseline corrected using ACD Labs 1D NMR processor. Spectra were then overlaid, the water and urea peaks excluded (4.5 – 6.0 ppm), and reduced to 12 Hz buckets (0.02 ppm) resulting in 376 integral regions. Each integral region was then normalized to the total peak area (0.01 – 9.02 ppm) in a constant sum method to limit the effects of concentration differences between the samples [16].

Spectral buckets were analyzed for pattern recognition using multivariate statistical analyses following Pareto scaling of the data [16]; specifically, principal components analysis (PCA) and orthogonal projection to latent structures discriminant analysis (OPLS-DA) using SIMCA P-13 (Umetrics). Coefficient parameters, *R^2^* (how much variation can be attributed to components [17]) and *Q^2^* (prediction accuracy, where scores greater than 0.6 are considered robust [17]) were examined to determine the most suitable model for further analysis. The supervised mode (OPLS-DA) was finally chosen for subsequent analysis as there was greater separation between the two data sets (pre- and post-exercise) for studying the variation in concentration of metabolites, and since the coefficient parameters were maximized in this case. Metabolites were identified using the Human Metabolites Database of the University of Alberta [18]. Approximations of relative changes in contributing metabolite concentration from pre- to post-exercise (fold change) were calculated using manually integrated peak areas and were reported as a mean fold change ± SD. Univariate significance testing (*p*<0.05) was conducted using an unpaired Student's *t*-test without multiple testing.

### Pathway Analysis

Differentially expressed transcripts or metabolites were functionally annotated using MetaCore Data-Mining and Pathway Analysis software (Thomson Reuters, NY, USA) to link significantly different genes or metabolites to databases of signaling networks and pathway maps. Briefly, for each pathway/network, the fraction of differentially altered genes or metabolites was compared to the total genes or metabolites representing that pathway/network, and the probability of the involvement of the respective number of altered genes or metabolites in that particular pathway/network was expressed as a *p*-value.

### Statistical Analysis

Statistical significance of all data was determined by unpaired *t* test, unless otherwise mentioned. Statistical analyses were performed using GraphPad Prism software and differences were considered statistically significant at *p*<0.05.

## Results

### Physiological Characteristics of Subjects

The physiological characteristics of all participants are presented in Table 1. Subjects in both control and athlete groups were appropriately matched for age, height, body weight, BMI and waist circumference, for the purpose of comparing the effects of long-term exercise training in older subjects on gene expression and metabolomics profiles. In addition, a panel of blood metabolic parameters was measured with nearly identical fasting blood glucose, hemoglobin, hematocrit, total and LDL cholesterol (Table 1). No difference in percentage of white blood cells (WBCs) was observed between the two groups at baseline (data not shown). The athlete group, however, had significantly lower fasting insulin levels (18.1±4.3 pM) versus control subjects (32.6±13.8 pM). In addition, the values for Homeostatic Model Assessment used to quantify insulin resistance (HOMA-IR), were also significantly lower in the athletes (0.61±0.16 vs 1.16±0.51), indicating that they were, in general, more insulin-sensitive than the control participants. Furthermore, the athletes displayed significantly elevated high density lipoprotein (HDL) with respect to the controls (1.91±0.49 vs 1.28±0.26 mM), accompanied by a significantly lower lipid ratio (3.0±0.6 vs 4.2±0.9). Overall, the metabolic measurements suggest a healthier lipid profile in the athlete group as compared to the control subjects.

**Table 1:** Physiological characteristics of all participants.

	Control	Athletes	p-value
Age (years)	54.3±5.0	53.4±3.2	0.69
Height (cm)	174±8	179±8	0.21
Weight (kg)	77.4±10.8	77.6±7.2	0.98
BMI (kg/m^2^)	25.4±3.2	24.1±2.5	0.36
Waist Circumference (cm)	91±7.2	84.4±5.5	0.06
Fasting Blood Glucose (mM)	5.4±0.4	5.3±0.3	0.54
Fasting Insulin (pM)	32.6±13.8	18.1±4.3^*^	< 0.01
HOMA-IR	1.16±0.51	0.61±0.16^*^	< 0.01
Triglycerides (mM)	1.30±0.46	0.98±0.37	0.16
Cholesterol (mM)	5.12±0.47	5.53±1.01	0.35
HDL Cholesterol (mM)	1.28±0.26	1.91±0.49^*^	< 0.01
LDL Cholesterol (mM)	3.25±0.41	3.19±0.72	0.86
Lipid Ratio	4.2±0.9	3.0±0.6^*^	< 0.01
Hemoglobin (g/L)	152±6	151±11	0.86
Hematocrit (L/L)	0.43±0.03	0.44±0.03	0.47
Absolute VO_2peak_(mL/min)	2803±812	4582±552^*^	< 0.01
Relative VO_2peak_ (ml·kg^−1^·min^−1^)	35.9±9.7	59.1±5.2^*^	< 0.01
HR_max_ (bpm)	179±6	173±9	0.33
Peak Aerobic Power (W)	230±57	383±39^*^	< 0.01
Sub-endurance Ride Time (min)	46.9±1.0	47.2±0.6	0.37
Respiratory Exchange Ratio	1.18±0.04	1.15±0.08	0.74

Values are expressed as mean ± SE. “*” indicates statistical significance; p-value<0.01

Analysis of peak oxygen consumption revealed significantly higher absolute and relative VO_2peak_ values in the athletes (4582±552 ml/min and 59.1±5.2 ml·kg^−1^·min^−1^) versus the control group (2803±812 ml/min and 35.9±9.7 ml·kg^−1^·min^−1^) and associated elevated peak aerobic power (383±39 W vs 230±57 W). No differences were observed between groups in maximal heart rate attained during the VO_2peak_ test or during the submaximal endurance ride. Overall, the data indicate a considerably better cardiometabolic and aerobic fitness profile in the athletes, compared to the control subjects.

### Gene Expression Analysis from Whole Blood

**Figure 1:**
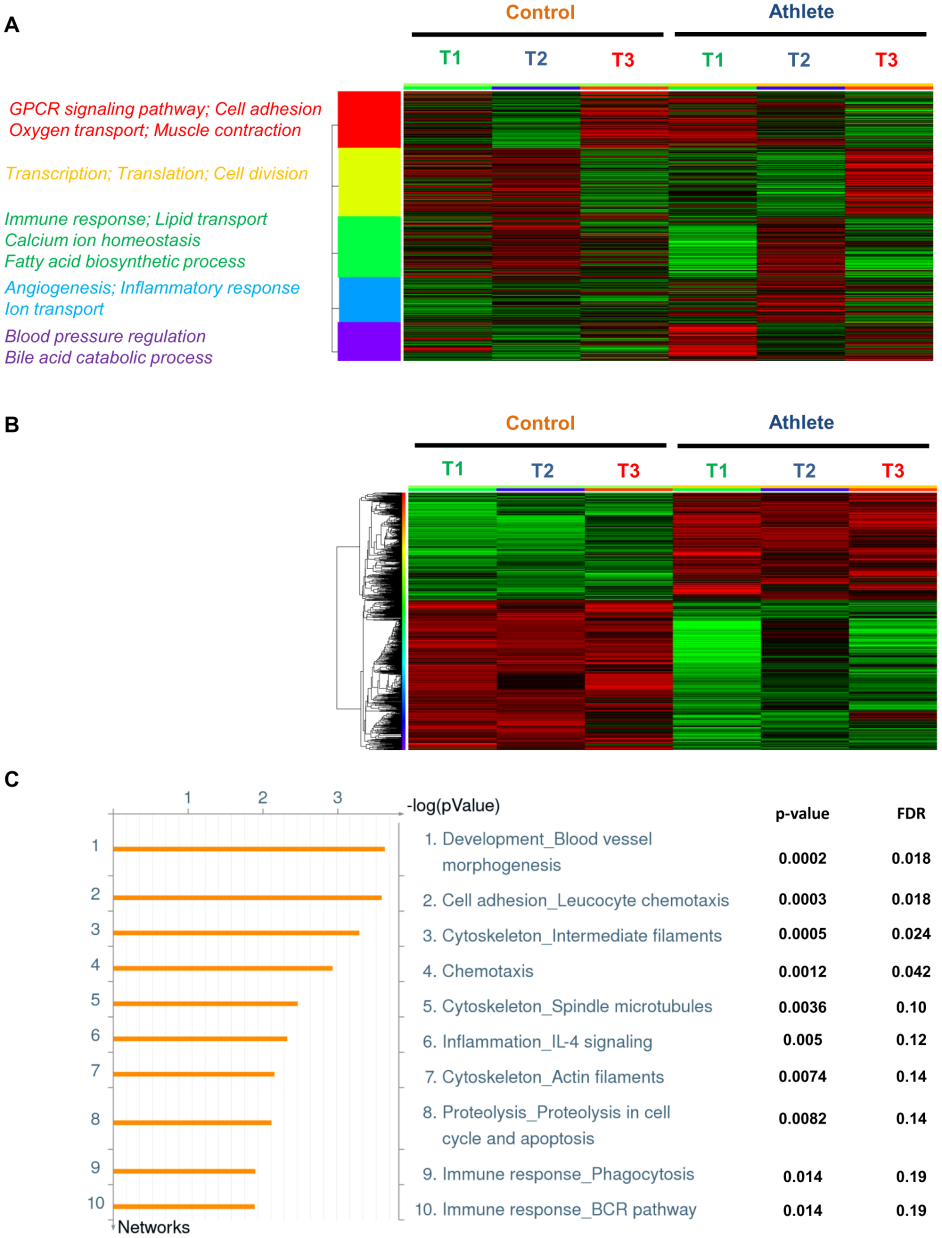
Gene expression in control and athlete subjects. (**A**) Heatmap of the entire genome including all normalized and filtered probes in athlete and control groups across all time-points (T1: 24 hrs before exercise; T2: immediately after exercise; T3: 24 hrs after exercise). Red represents an up-regulation, and green a downregulation in gene expression. The probes were clustered by k-means clustering into 5 broad clusters. Representative Gene Ontology (GO) biological processes of each cluster are shown in their respective colours on the left. (**B**) Representative heatmap of probes found significantly different between athlete and control groups across all time-points (T1: 24 hrs before exercise; T2: immediately after exercise; T3: 24 hrs after exercise). The probes were clustered by hierarchical clustering. Fold change>1.5; False Discovery Rate (FDR)<0.1. (**C**) Top 10 networks representing genes differentially expressed between athlete and control groups across all time points by Metacore analysis, with corresponding *p*-values and FDR.

To determine the pattern of gene expression in athlete and control groups at the three different time-points (T1: before exercise; T2: immediately after exercise; T3: 24 hours after exercise), we carried out whole blood transcriptome microarray analysis using Agilent's 4 × 44 K array chips. After quantile normalization of the data and filtering out probes with variance greater than 0.1 across samples, of the 41,077 probes represented on the array, a total of 28,709 probes were used for further analysis. A distribution in expression of these probes representing the entire genome in athlete and control groups across all time-points is represented as a heatmap (Figure 1A). k-means clustering was further used to group the probes into 5 main clusters in order to better elaborate the variation in their distribution across the data-set. An enrichment analysis to enquire about what these 5 clusters meant in biological terms led to the identification of a host of biological processes, a few of which are represented beside each cluster. Thus, Figure 1A illustrates that considerable variability exists in genome-wide expression between the athlete and control groups before (T1) and after (T2 and T3) exercise.

A direct comparison analysis to detect statistically significant differential probes led to the identification of 648 probes representing 393 genes which were differentially regulated between the athlete and control groups across all the time-points combined. This analysis was based on stringent statistical parameters involving a fold change of > 1.5 for each probe, and a false discovery rate (FDR) threshold set to 10%, following multiple testing correction by Benjamini-Hochberg analysis. Figure 1B represents an illustrative heatmap of these differentially expressed genes, arranged to two hierarchical clusters between the two groups across all time-points. A list of all these differentially expressed probe sets is provided in Table S1. To interpret their biological significance, we also carried out an annotation analysis (see Methods section) to classify them to functional relevant categories. Figure 1C represents the top ten networks interrogated by this dataset, with their corresponding *p*-values and FDRs. While “blood vessel morphogenesis” emerged as the top network, a number of immune response networks, including leukocyte chemotaxis were also found to be differentially regulated. The other top networks included inflammation through IL-4 signaling, as well as a host of cytoskeleton reorganization networks.

**Figure 2:**
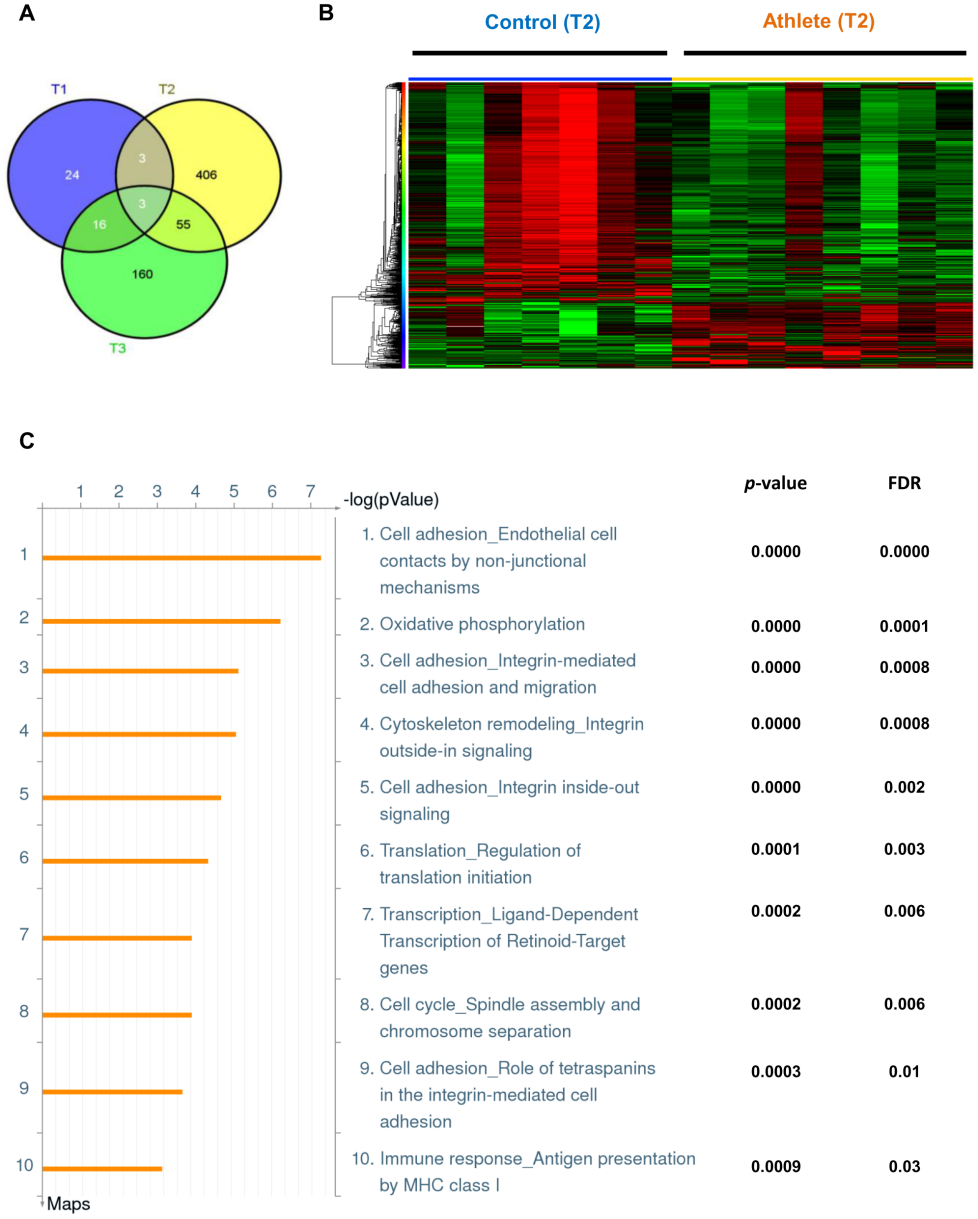
Differential gene expression between control and athlete subjects after exercise. (**A**) Venn diagram representing genes differentially expressed between control and athlete groups at time points T1 (blue; 24 hrs before exercise; n = 4 for each group); T2 (yellow; immediately after exercise; n = 7 for controls and n = 8 for athlete group); and T3 (green; 24 hrs after exercise; n = 4 for each group). Fold change>1.5; False Discovery Rate (FDR)<0.1. Genes differentially regulated at two or three time points are shown in the corresponding intersecting segments. (**B**) Heatmap representing genes differentially expressed between individual control and athlete samples at time point T2 (immediately after exercise). Each column represents an individual subject. Red represents an up-regulation, and green a down-regulation in gene expression. Probes and samples are clustered by hierarchical clustering Fold change>1.5; FDR<0.1. (**C**) Top 10 canonical pathway maps representing genes differentially expressed between athlete and control groups at time-point T2 by Metacore analysis with corresponding *p*-values and FDR.

In order to identify which time-point of exercise was contributing most to the differential gene expression between the athlete and control groups, we performed individual comparison analyses between the two groups at each time-point, the results of which are shown in the form of a Venn diagram in Figure 2A. Only 73 probes representing 46 genes were found to be differentially regulated (fold change>1.5; FDR<10%) between the athlete and control groups before exercise at time-point T1 (entire list of differential genes provided in Table S2). In contrast, at time-point T2, immediately after exercise, a total of 691 probes, representing 467 genes were found to be differentially regulated (fold change>1.5; FDR<10%) between the two groups (Table S3). Finally, at time-point T3, 24 hours after exercise, 385 probe-sets representing 234 genes were altered (fold change>1.5; FDR<10%) between the athletes and controls (Table S4). As illustrated in Figure 2A, comparing genes significantly different between the athlete and control groups, 3 genes were found to be altered at both time-points T1 and T2, 16 genes at both T1 and T3, and a total of 55 genes for both T2 and T3 time-points. Combining all the time-points, only 3 genes (*SLC5A11*, *OLFM4*, *LPIN1*) were found to be similarly altered between athlete and control groups at all three time-points. A list of these similarly regulated genes is provided in Table S5. Thus, of all the three time-points, time-point T2, immediately after exercise, exhibited the most number of genes which were significantly altered between the athlete and control groups. This led us to focus primarily on time-point T2 for further analysis of differential gene expression between the two groups.

Out of 691 differentially expressed probe sets between the two groups at T2, 160 were up-regulated, while 531 were down-regulated in athletes compared to control subjects (Table S3). Figure 2B represents a heatmap illustrating the differential expression of these 691 probes (467 genes) across each individual sample. A two-way hierarchical clustering analysis of both probe-sets and samples revealed that seven of eight athlete samples exhibited striking similarity in gene expression and clustering. Likewise, a distinct gene expression pattern was observed amongst six of seven control samples, with one control subject being an outlier and clustering with the athlete group.

Next, an enrichment analysis was carried out as described in the Methods to functionally annotate the differential genes into matching gene ontology pathway maps. Figure 2C represents the top ten pathway maps, with their corresponding *p*-values and FDR, representing the differential gene list between athlete and control groups at time-point T2. We found ‘oxidative phosphorylation’ as one of the top ranked pathways to be regulated for this data-set, along with various cell adhesion and cytoskeleton remodeling pathways. Additional pathways altered included immune response, as well as transcription and translation.

Of the 467 genes differentially regulated between athletes and controls at time-point T2, we selected a representative subset of genes, with the highest fold change values, the regulation of which are known to have important cardiovascular, metabolic and other disease relevance (Table 2). A detailed description of a few of these genes and their biological significance is provided further in the Discussion section. Table 2 outlines these genes with their corresponding fold changes and *p*-values. A negative fold change signifies down-regulation, while a positive fold-change represents up-regulation in athletes compared to control subjects. To increase the accuracy of our findings and validate our results, we utilized a second microarray analysis software (GeneSpring, Agilent Technologies) to confirm the regulation of genes outlined in Table 2. Analysis by GeneSpring revealed similar direction of regulation of all the selected genes with nearly identical fold changes as carried out using the Nexus analysis software.

**Table 2:** List of differentially expressed genes.

*Probe ID*	*Gene Symbol*	*Gene Name*	*Fold change (Nexus)*	*q-value (Nexus)*	*Fold Change (Genespring)*	*Corrected p-value (Genespring)*
**A_23_P63343**	***UTS2***	**Urotensin 2**	**−4.76**	**0**	**−5.42**	**0.044**
A_24_P28977	*TRPC1*	Transient receptor potential cation channel, subfamily C, member 1	−4.06	0	−4.94	0.042
A_23_P210100	*CYP26B1*	Cytochrome P450, family 26, subfamily B, polypeptide 1	3.6	0.005	5.22	0.042
**A_23_P92672**	***OCLN***	**Occludin**	**2.66**	**0.0005**	**3.67**	**0.041**
A_23_P31671	*UQCRB*	Ubiquinol-cytochrome c reductase binding protein	−2.65	0.0002	−2.93	0.049
A_24_P231104	*LEPR*	Leptin receptor	−2.55	0.01	−2.71	0.041
A_32_P134056	*DOCK4*	Dedicator of cytokinesis 4	2.43	0.0006	3.02	0.048
**A_23_P63209**	***HSD11B1***	**Hydroxysteroid (11-beta) dehydrogenase 1**	**−2.35**	**0.008**	**−2.56**	**0.044**
A_24_P257224	*TPO*	Thyroid peroxidase	−2.26	0.002	−2.36	0.041
A_23_P150609	*IGF2*	Insulin-like growth factor 2/somatomedin A	2.24	0.001	2.47	0.048
**A_23_P102454**	***INSIG2***	**Insulin induced gene 2**	**−2.07**	**0.002**	**−2.21**	**0.047**
A_32_P153071	*VIPR2*	Vasoactive intestinal peptide receptor 2	2.02	0.009	2.26	0.045
**A_23_P205986**	***IGF1R***	**Insulin-like growth factor 1 receptor**	**1.38**	**0.025**	**1.5**	**0.046**

Representative list of genes differentially expressed between control and athlete groups at time point T2. Those indicated in bold have been validated by quantitative real-time PCR.

**Figure 3:**
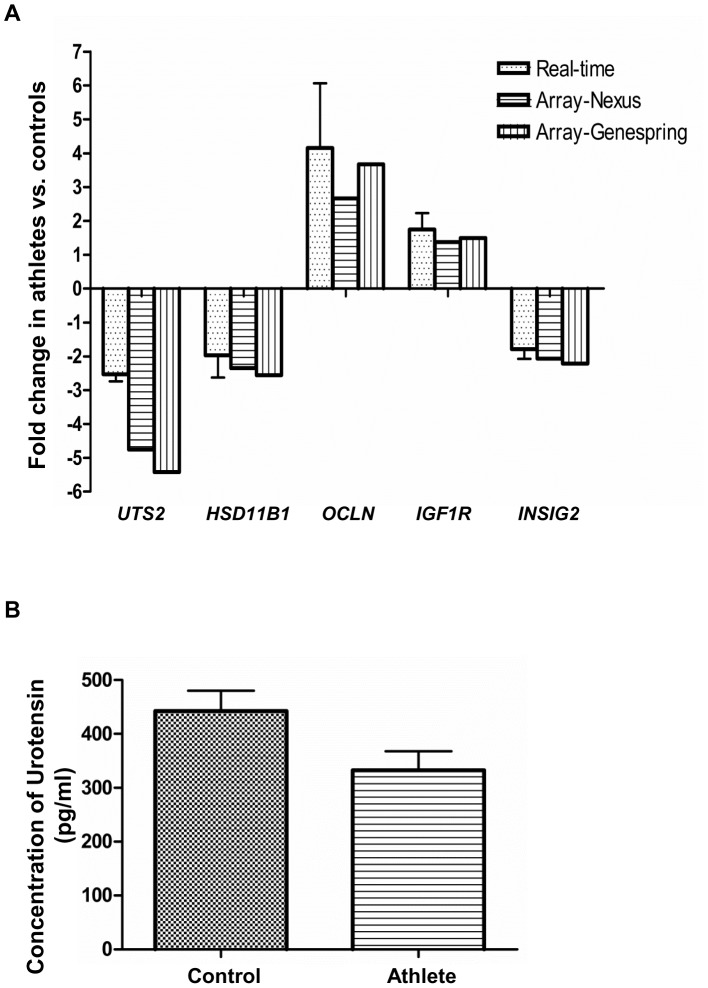
Validation of microarray data. (**A**) Fold-change in expression of genes *UTS2*, *HSD11B1*, *OCLN*, *IGF1R* and *INSIG2* between athlete (n = 8) and control (n = 7) groups at time point T2, as evident by microarray analysis (Nexus Gene Expression and Genespring), and validated by quantitative real-time PCR (qRT-PCR). qRT-PCR data are normalized to the housekeeping gene and were statistically significant with *p*<0.05 (n = 6 in each group). (**B**) Concentration of urotensin (pg/ml) in the plasma of control (n = 8) and athlete (n = 6) subjects at time-point T1.

To further validate the accuracy of our microarray data, we examined the relative expression of five genes (*UTS2, HSD11B1, OCLN, IGF1R* and *INSIG2*) differentially expressed between athlete and control groups at time-point T2, by quantitative real-time PCR (qPCR) (Figure 3A). Expression patterns, as evident by fold changes, were similar between the microarray findings (both Nexus and GeneSpring analyses) and the qRT-PCR measures for all the genes measured with statistical significance achieved in qRT-PCR (*p*-value<0.05). Furthermore, we were able to validate both up-regulated, as well as down-regulated genes by qRT-PCR. In addition, we examined the concentration of urotensin in the plasma obtained from control and athlete subjects at time-point T1 by ELISA. Although it did not reach statistical significance, we could see a clear trend in decreased urotensin concentration in the athletes compared to the control subjects (*p*-value = 0.06).

### Metabolomic Analysis of Urine by NMR Spectroscopy

**Figure 4:**
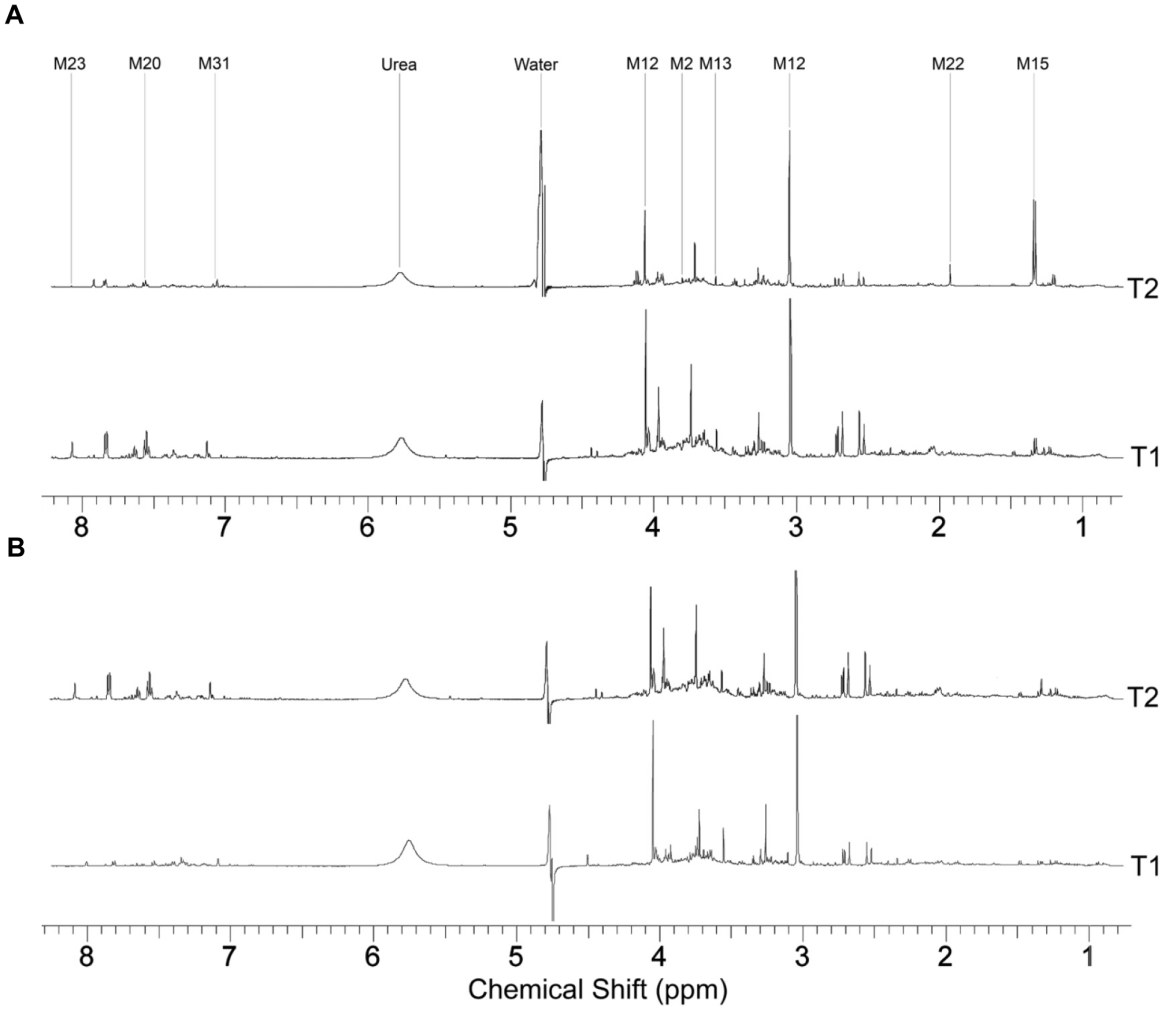
^1^H NMR spectra of control and athlete urine samples. ^1^H NMR spectra of a representative pre- (T1) and post-exercise (T2) urine sample from an athlete (**A**) and control (**B**) subject highlighting visible differences between the two spectra and the major resonances. Numbered biomolecules correspond to Table 3.

Representative ^1^H-NMR spectra of pre- and post-exercise urine samples are shown in Figure 4. Major resonances identified in the spectra consistent over both time points were assigned to creatinine (3.05, 4.03 ppm), citrate (2.53 and 2.65 ppm), TMAO (3.25 ppm) and glycine (3.54 ppm). The major differences in component peaks between the time-points are identified in Table 3 and Figure 4. All identified metabolite component resonances are shown in Table S7. Visual inspection is a limited method of comparison for use with NMR spectra obtained from urine specimens due to the level of complexity of signals and varying dilution of the samples.

**Table 3:** 1H NMR of urine metabolites.

*Metabolite number*	*Name of metabolite*	*δ (ppm)*	*Athlete Fold Change (±SD)*	*Control Fold Change (±SD)*	*p-value*
M1	2-Hydroxybutyrate	0.90	1.28±0.23	1.48±0.65	0.44
M2	2-Hydroxyisovalerate	3.85	1.12±0.39	1.44±0.12	0.046*
M3	α-Ketoglutarate	2.45	0.98±0.29	1.03±0.41	0.78
M4	2-Oxoisocaproate	0.94	1.19±0.12	1.24±0.12	0.40
M5	2-Oxoisovalerate	1.12	1.28±0.39	1.31±0.59	0.94
M6	3-Hydroxybutyrate	1.20	0.82±0.21	0.77±0.19	0.64
M7	3-Hydroxyisobutyrate	1.07, 3.68‡	1.33±0.24	1.38±0.35	0.78
M8	3-Methyl-2-oxovalerate	1.10, 2.94‡	1.11±0.19	1.15±0.35	0.82
M9	Acetoacetate	2.24	0.91±0.12	1.05±0.26	0.23
M10	Alanine	1.49	1.16±0.29	1.12±0.39	0.83
M11	Citrate	2.55	0.63±0.15	0.66±0.19	0.70
M12	Creatinine	3.05	1.03±0.21	1.14±0.22	0.34
M13	Glycine	3.57	0.84±0.09	0.93±0.04	0.019*
M14	Formate	8.46	0.96±0.05	0.84±0.14	0.043*
M15	Lactate	1.33	4.34±1.21	7.16±6.61	0.10
M16	Malonate	3.11	1.13±0.35	1.78±0.41	0.004*
M17	Phenylalanine	3.98, 7.36‡	0.91±0.09	0.95±0.11	0.49
M18	Pyruvate	2.35	1.70±0.22	1.68±0.43	0.92
M19	Succinate	2.53	0.89±0.12	0.81±0.19	0.12
M20	Hippurate	7.56	0.91±0.56	0.96±0.63	0.86
M21	Trimethylamine N-oxide	3.25	0.88±0.22	0.83±0.31	0.70
M22	Acetate	1.93	1.32±0.14	4.21±0.12	0.0001*
M23	Hypoxanthine	8.19	4.34±1.12	2.91±0.98	0.016*
M24	Tryptophan	4.06, 7.32‡	0.83±0.05	0.94±0.08	0.004*
M25	Inosine	4.28‡, 6.10	1.39±0.71	1.29±0.84	0.81
M26	Glucuronate†	3.73	-	-	-
M27	3-Hydroxyisovalerate†	1.26	-	-	-
M28	Valine	0.99, 3.60	0.98±0.07	1.01±0.12	0.55
M29	Fumarate	6.51	2.16±0.45	1.53±0.39	0.009*
M30	Malate♮	4.29	-	-	-
M31	Histidine♭	7.09	-	-	-
M32	Leucine♮	0.95, 1.70	-	-	-

1 H NMR identified urine metabolites and relative changes in concentration reported as fold change for athlete and control groups after exercise.

‡Signal not used for concentration calculation.

†Peak areas were unreported due to peak overlap.

♮Metabolite not detected.

*Significant difference between gr°ups.

♭Histidine concentrations were not calculated due to dynamic nature of His resonances.

**Figure 5:**
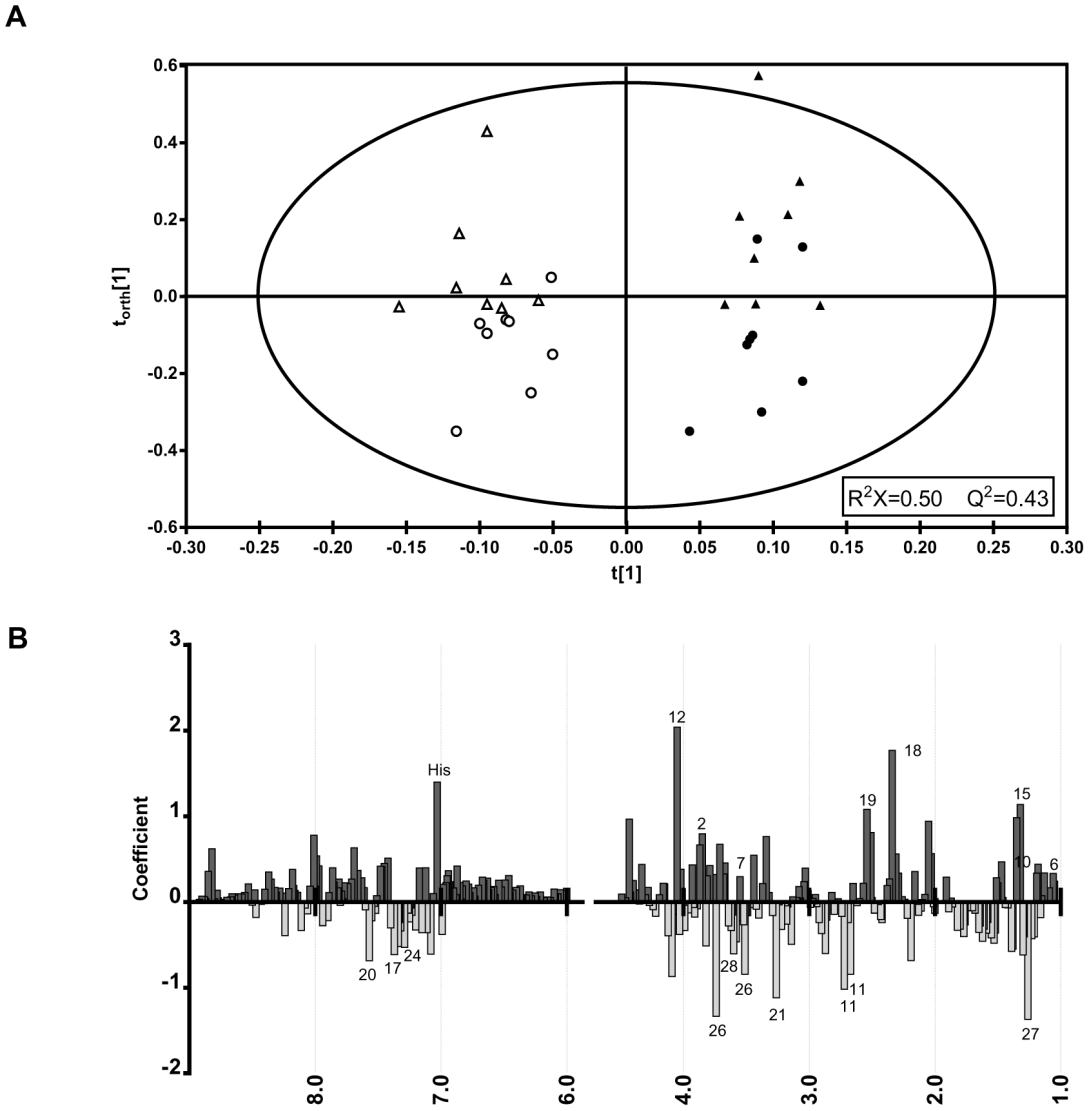
Statistical analysis of ^1^H NMR spectra. (**A**) OPLS-DA component plot derived from spectra of urine before (▴, athlete and •, control) and after execise (Δ, athlete and ○, control). R^2^X = 0.50; Q^2^ = 0.43. (**B**) OPLS coefficient plot showing the most significant contributors to the separation. Numbered biomolecules correspond to Table 3.

Thus, further comparison of the spectra was conducted using the multivariate pattern recognition technique, PCA, which revealed no clear distinction between the athlete and control groups [*R^2^* = 0.49, *Q^2^* = 0.12]. Since the inherent metabolic variation between the two time-points was not sufficient to separate pre- and post-exercise samples, OPLS discrimination analysis was conducted using the constant sum normalized spectra [*R^2^X* = 0.50, *Q^2^* = 0.43]. PCA and OPLS-DA scores for constant sum and creatinine normalization methods are shown in Table S8. The reliability of the model was verified using a cross-validated OPLS-DA score map. Using the OPLS-DA method, the variation associated with the time-point was supervised, focusing the model on intergroup variability. The two groups were more clustered than in the unsupervised model (OPLS-DA *Q^2^* = 0.43; PCA *Q^2^* = 0.12); however, there was still some overlap between the athletic and control participants (Figure 5A).

To identify which chemical shifts were accountable for intergroup variability, contribution analysis was conducted using a coefficient plot, which highlighted the chemical shifts (δ) of maximal variation (Figure 5B), indicated by the magnitude of the coefficient (VIP score). The most significant of these are lactate (δ = 1.33 ppm, VIP = 1.14), 3-hydroxisovalerate (δ = 1.26 ppm, VIP = −1.37), pyruvate (δ = 2.35 ppm, VIP = 1.77), glucuronate (δ = 3.73 ppm, VIP = −0.88), creatinine (δ = 4.05 ppm, VIP = 2.04), and histidine (δ = 7.03, VIP = 1.4).

Following the identification of major contributing biomolecules in the OPLS-DA model, metabolites were quantified by direct integration and constant sum normalized. Fold-change values were calculated for each using identifying peaks shown in Table 3. Unpaired Student's *t*-tests indicated that acute exercise had a significantly different impact on excretion concentrations of 2-hydroxyisovalerate (p = 0.046), acetate (p = 0.0001), malonate (p = 0.004), hypoxanthine (p = 0.016), formate (p = 0.043), glycine (p = 0.019), fumarate (p = 0.009), and tryptophan (p = 0.004) between the athlete and control groups.

**Figure 6:**
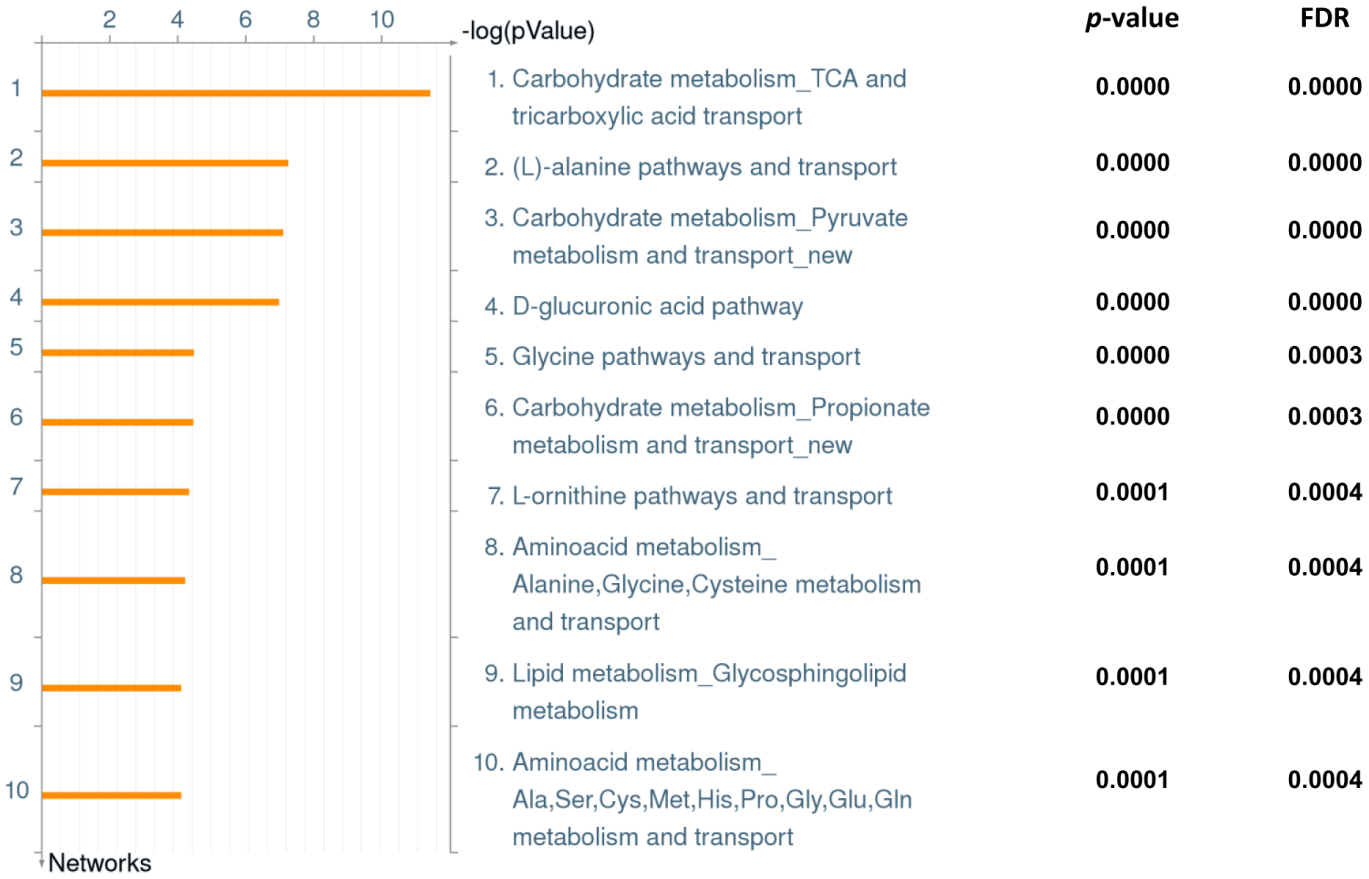
Pathway analysis of urinary metabolites. Top 10 networks representing metabolites differentially expressed between athlete and control groups pre- and post-exercise by Metacore analysis, with corresponding *p*-values and FDR.

Figure 6 presents the top ten differential networks that emerged from the univariate analysis data with corresponding *p*-values and FDR. ‘Carbohydrate metabolism, TCA and tricarboxylic acid transport’ was found to be the top ranked differential pathway between the two groups. Others include various types of amino acid and transport systems and lipid metabolism.

## Discussion

Regular exercise reduces the risk of a variety of cardiovascular, metabolic, musculoskeletal and neurological disorders typically associated with aging [1]. Aging is a multi-faceted phenomenon integrating changes across cells, tissues and whole body systems making the study of aging complex and necessitating the use of multiple methodologies and platforms. In an attempt to overcome this complexity, recent reports have advocated the use of a systems biology approach to integrate genome-, proteome- and metabolome-wide data sets for a deeper and broader understanding of the aging process [19], [20]. A similar approach has been suggested to study physiological adaptation to endurance exercise [21], with multiple studies emerging in the last decade using data-sets from different platforms to define exercise-induced adaptation [11], [12], [22], [23]. Thus, the aim of our current study was to utilize a dual approach using genome-wide microarray and NMR-based metabolomics to elucidate the molecular details of the exercise response in aging, 50–60 year old elite Masters athletes.

### The Blood Transciptome: Impacts of Training Status

Although several studies have reported the use of microarray analysis to detect exercise-induced changes in gene expression, these analyses are largely limited to non-athlete young adults [2]–[5]. The few studies that have examined athletes studied a much younger age group than the current investigation and probed specifically into subsets of stress or immune response genes using custom-made cDNA microarrays [6], [7]. To our knowledge, the current study is the first to compare the genome-wide change in whole blood gene expression in aged (50 – 60 years old) competitive Masters athletes and untrained individuals. Importantly, we have utilized whole blood to analyze these changes as opposed to skeletal muscle biopsies or PBMCs, which represent only a fraction of WBCs in whole blood. The whole blood transcriptome possesses >80% expression similarity to several tissues of the human body, with “residual genetic footprints” as a consequence of cellular changes in other tissue types [10]. Moreover, alterations in gene expression in skeletal muscle adaptation to endurance training are significantly correlated to changes in blood cell gene expression [24].

Our analysis revealed a subset of 393 genes significantly different between the athlete and control groups across all time-points combined. A pathway and network analysis revealed that “blood vessel morphogenesis” was the most significantly different network of genes between the athlete and control groups. Consistent with this difference in whole blood gene expression, angiogenesis is known to result from exercise training and results in improved capillarization, oxygen exchange and blood flow capacity in skeletal muscle [25], [26], and may contribute to exercise mediated improvements in cognitive function [27], [28]. The second ranked network significantly altered was the “cytoskeleton” involving “intermediate filaments”, “spindle microtubules” and “actin filaments”. This finding may also reflect the physiological impact of exercise training as skeletal muscle structure and composition change with advancing age [29], while moderate and continuous exercise training can lead to remodeling of skeletal muscle ultrastructure, subsequently increasing endurance capacity [30]. Similar results have also been reported in a transcriptome study of equine skeletal muscle response to exercise training [31]. Additional networks differentially regulated between the athlete and control groups include anti-inflammatory “IL-4 signaling” pathway and “immune response” involving both adaptive and innate immunity, which are protected from age-related senescence by exercise [32]–[34].

Analysis of changes in gene expression between the control and athlete groups at time-points T1, T2 and T3 led to the identification of three genes (*SLC5A11, OLFM4, LPIN1*), which were differentially regulated between the two groups at all three time-points (Table S5). *SLC5A11* (solute carrier family 5 [sodium/inositol cotransporter], member 11) is a sodium dependent glucose (myo-inositol) transporter [35], with higher expression in control subjects possibly indicating the necessity to pump more myo-inositol into their cells in an attempt to increase insulin sensitivity [36]. Expression of *OLFM4* (olfactomedin 4), an antiapoptotic factor known to act as a marker for colorectal and gastric cancer [37], [38], was also decreased in athletes, perhaps denoting a potential benefit of exercise training against these tumor types. On the other hand, the athlete group displayed an increased expression of *LPIN1* (Lipin 1), the most important member of the lipin family of proteins known to regulate triglyceride and phospholipid metabolism [39]. Both human and mouse studies have reported positive correlation of lipin-1 expression and enhanced insulin sensitivity and glucose metabolism in healthy men [40], [41], findings that are consistent with our demonstration of improved insulin sensitivity in the athlete group (Table 1). Interestingly, enhanced expression of lipin-1 is also involved in exercise-induced mitochondrial biogenesis in skeletal muscle [42]. Combined, these results suggest that the upregulation of lipin-1 may contribute to the enhanced insulin sensitivity and exercise capacity observed in the veteran athletes in the current study.

### Acute Changes in the Blood Transcriptome Following Exercise

Of the three time-points analyzed, we have focussed on T2, for further analysis. This decision was driven by the relative abundance of genes that were differentially regulated between the two groups at T2 (467 genes), compared to T1 (46 genes) and T3 (234 genes). Of particular interest is the finding that the number of genes differentially expressed across different time points (T1 vs. T2; T2 vs. T3; T1 vs. T3; Table S6) was considerably higher in the athlete group. In addition, pathway analysis revealed that genes associated with the “oxidative phosphorylation” and “cell adhesion-integrin” pathways were among the top pathways altered differentially between groups. Given the relationship between oxidative phosphorylation and aerobic capacity/performance [43] and between “cell adhesion-integrin” pathways and vascular adaptations to exercise [44], these changes, combined with the greater overall change in gene expression following exercise may reflect a greater adaptive response to exercise in athletes, potentially explaining the maintenance of elevated aerobic capacity despite their advancing age. Also of note is the relative homogeneity of the within group responses (T2 analysis revealed that 7 out of 8 athletes and 6 of 7 control subjects clustered in distinct gene expression patterns; Figure 2B). This similarity in the within group responses to exercise further strengthens the idea that specific markers within the blood transcriptome may be reliable markers of both metabolic and cardiovascular health and fitness, and may provide future diagnostic value in clinical settings.

We identified a small subset of genes of interest for further analysis with high fold changes between the athlete and control groups at time-point T2, which are involved in a variety of cardiovascular, metabolic, neurological and other age-related functions and disorders (Table 2). *UTS2* (Urotensin 2), the most potent endogenous vasoconstrictor, is widely expressed in blood cells and the cardiovascular system, and displays smooth muscle-dependent vasoconstriction and influences proliferation of smooth muscle cells, as well as chemotaxis of inflammatory cells [45]. Moreover, urotensin 2 inhibits insulin release, has inotropic and hypertrophic effects on heart muscle, and is a proposed biomarker of cardio-metabolic disorders including hypertension, atherosclerosis, heart failure, pulmonary hypertension, diabetes, renal failure and metabolic syndrome [45]. Thus, our current microarray findings suggest a potential cardio-metabolic benefit in the athletes with significantly lower *UTS2* expression compared to the control group at T2 (Table 2), which was further confirmed by real-time PCR (Figure 3A). In addition, we also found a decreased urotensin concentration in the plasma of athletes before exercise, compared to the control subjects (Figure 3B). *HSD11B1* (Hydroxysteroid (11-beta) dehydrogenase 1) was also significantly down-regulated in the athletes (Table 3 and Figure 3A). HSD11B1 is a bidirectional microsomal enzyme that interconverts stress hormone cortisol and the inactive metabolite cortisone [46]. HSD11B1 is well-known to play a pathogenic role in obesity, the metabolic syndrome and type 2 diabetes mellitus, and HSD11B1 inhibitors are currently being studied in both animals and humans as possible pharmacotherapies [47]. Our data with lower *HSD11B1* expression in athletes are consistent with these findings. Occludin (OCLN) is a major component of tight junctions, which play critical roles in barrier function between cellular compartments [48]. Alterations in the regulation of occludin and consequent disruption of tight junctions have been associated with a variety of age-associated diseases such as inflammatory bowel disease, multiple sclerosis, cancer, diabetes and allergic disorders [48], [49]. However, it has recently been reported that exercise can protect the blood-brain barrier from ischemic injury by inhibiting the reduction of occludin in rats [50]. Our current results, which show a significantly higher expression of *OCLN* in the athletes than the control subjects, suggest a potential protection against gastrointestinal and other geriatric dysfunctions in the aged athletes.

Other genes down-regulated in the control group include *VASN* (vasorin), expressed in smooth muscle cells, the deficiency of which may lead to vascular fibroproliferative disorders [51], *DOCK4* (Dedicator of cytokinesis 4) and *NRXN1* (Neurexin 1), dysregulation of which lead to autistic disorders and schizophrenia [52], [53], *VIPR2* (Vasoactive Intestinal Peptide Receptor 2), known to provide neuroprotection and currently being studied as a candidate gene for schizophrenia [54], *MYH4* (Myosin heavy chain 4) whose dysregulation causes skeletal muscle myofibrillar degeneration [55], *IGF2* (Insulin-like growth factor 2), related to memory enhancement [56] and *IGF1R* (Insulin-like growth factor 1 receptor), which regulates functions of insulin, a decrease of which can lead to metabolic disorders [57]. In contrast, genes significantly increased in the control subjects include *TRPC1* (Transient receptor potential cation channel, subfamily C, member 1), which is associated with a wide variety of cardiovascular diseases including neointimal hyperplasia, hypertension, atherosclerosis and hypertrophy [58], *UQCRB* (Ubiquinol-cytochrome c reductase binding protein), a subunit of ubiquinol-cytochrome c oxidoreductase complex involved in oxidative phosphorylation [59] and *INSIG2* (Insulin-induced gene 2), which is overexpressed in a variety of colon and pancreatic cancers [60], [61]. Overall, our genome-wide microarray analysis revealed a network of candidate genes in various functional and signaling pathways, including those mentioned above, differentially expressed between the control and athlete groups, which provides the framework for targeted questions on defining adaptive pathways of healthy aging and the use of the blood transcriptome as a potential diagnostic tool in exercise physiology.

### Urine Metabolomics, Fitness and Aging

NMR-based metabolomics is a semi-quantitative method of analyzing biofluids, which is useful in determining effects of exercise on both group, and individual levels [13], [14]. We have focused on the relative differences in concentration between the athlete and control groups for detectable metabolites which were expected to respond differently to acute exercise. Multivariate analysis revealed that there are vast differences in urine metabolomics profiles 24 h before and 24 h after a submaximal endurance cycling test. Moreover, there are sufficient differences between the control and athlete groups during both time periods using OPLS-DA.

The excretion of a number of metabolites was different in the post-exercise period compared to pre-exercise. For example, lactate excretion was elevated ∼4 and ∼7 fold in the 24 hours post-exercise in the athlete and control groups respectively (difference between groups approached significance, *p*-value = 0.1). Between group differences in several other metabolites are consistent with differences in exercise metabolism expected between two groups with significant differences in fitness and training status. For example, acetate, which is formed in a radical-removing reaction of hydrogen peroxide [62], [63], was excreted to a greater extent in the control group, a finding that is consistent with reduced oxidative stress in well trained individuals [64]. Group differences in malonate (greater excretion in control) and fumarate (greater excretion in athletes) are also consistent with expected differences in succinate oxidation and overall TCA cycle turnover between groups [65], while greater excretion of hypoxanthine by athletes is consistent with the greater ATP turnover required to complete the higher absolute work performed by the athletes [66]. The greater increase in formate excretion in the control group may also reflect decreased NAD^+^ availability during exercise [14], another finding that is consistent with the between group differences in fitness and training status. Interestingly, there were also differences in the amount of amino acids excreted post-exercise between groups, and while these differences may be consistent with elevated amino acid degradation observed during exercise [67], the reasons for the between group differences are currently unclear. These results indicate that changes in the urine metabolome appear to reflect expected changes in whole body exercise metabolism, and may be utilized as a diagnostic marker for exercise, as well as provide targets for new pharmacological approaches that mimic the effects of exercise (“gymnomimetics”) [68].

### Summary and Conclusions

Thus, our current findings utilizing genome-wide microarray and NMR spectroscopy-based metabolomics approaches show that highly trained competitive athletes have a characteristic genetic/metabolic footprint after exercise that can be distinguished from non-trained, minimally active older individuals. These results warrant future studies into the signaling pathways of some of these genes and metabolites, which could lead to a better understanding of the molecular basis of healthy aging. Overall, these findings support the use of microarray and metabolomics approaches towards development of potential biomarkers for monitoring exercise and training load and thereby provide a relatively non-invasive means of tracking training status.

## Supporting Information

Table S1 **List of differentially expressed probes between athlete and control subjects across all time-points.**
(XLSX)

Table S2 **List of differentially expressed probes between athlete and control subjects at time-point T1 (before exercise).**
(XLSX)

Table S3 **List of differentially expressed probes between athlete and control subjects at time-point T2 (immediately after exercise).**
(XLSX)

Table S4 **List of differentially expressed probes between athlete and control subjects at time-point T3 (24 hours after exercise).**
(XLSX)

Table S5 **List of genes similarly regulated between athlete and control groups at different time-points.**
(XLSX)

Table S6 **Differentially expressed probes between different time-points in control and athlete groups.**
(XLSX)

Table S7 **1H NMR identified urine metab°lites and all respective chemical shift values.**
(XLSX)

Table S8 **Multivariate analysis PCA and OPLS-DA R^2^ and Q^2^ parameters and associated models.**
(XLSX)
